# The impact of biotic and abiotic interactions on *Candidatus* Kouleothrix bulking in a full-scale activated sludge anaerobic-anoxic-oxic plant in Japan

**DOI:** 10.1038/s41598-025-98211-9

**Published:** 2025-05-06

**Authors:** Tadashi Nittami, Nagi Ishizuka, Yoshiki Sakurai, Robert J. Seviour

**Affiliations:** 1 Division of Materials Science and Chemical Engineering, Faculty of Engineering, Yokohama National University, 79-5 Tokiwadai, Hodogaya-ku, Yokohama, 240-8501 Japan; 2 Department of Chemistry and Life Science, Graduate School of Engineering Science, Yokohama National University, 79-5 Tokiwadai, Hodogaya-ku, Yokohama, 240-8501 Japan; 3 Division of Artificial Environment and Information, Faculty of Environment and Information Sciences, Yokohama National University, 79-7 Tokiwadai, Hodogaya-ku, Yokohama, 240-8501 Japan; 4 Department of Physiology, Anatomy, and Microbiology, La Trobe University, Bundoora, VIC 3086 Australia

**Keywords:** “*Candidatus* Kouleothrix”, Eikelboom type 1851, Activated sludge bulking, Filamentous bacteria, Fluorescence in situ hybridization (FISH), Amplicon sequencing, Water microbiology, Applied microbiology, Microbial ecology

## Abstract

The genus “*Candidatus* Kouleothrix” (Eikelboom type 1851) is the major filamentous bacterium responsible for activated sludge bulking in Japanese activated sludge plants, where it is present in many in unusually high abundances. Global surveys have shown this genus embraces several species, although the contribution each of these makes to bulking is not known. This study followed their population dynamics in a full-scale bulking sludge plant in Japan over a 12-month period, which required the development of species-specific FISH probes, used to measure their relative abundances. Six of the 9 recognised species of “*Ca*. Kouleothrix” were detected there, two of which (midas_s_3423 and midas_s_35412) were at consistently high abundances and responsible for increases in the sludge volume index (SVI). Their abundances were also statistically correlated with their filament lengths, which showed higher correlations with SVI increases than did species abundances. This study examined the impacts of both operational parameters and other communities on the abundances of these two species over the 12-month period and showed marked interspecies differences in responses to both. Together these findings stress the importance in studies of this kind to identify populations to species level to reveal possible important ecological differences not seen at the genus level.

**Supplementary Information:**

The online version contains supplementary material available at 10.1038/s41598-025-98211-9.

## Introduction

Although still the most popular choice globally for the treatment of both municipal and industrial wastewaters^1^, the activated sludge process has undergone modifications in response to more stringent discharge standards required for final treated effluents^2^. Even so, inadequate secondary settling tank liquid phase separation still remains a serious unresolved operational issue^3,4^, which leads to poor solids/liquids separation and thickening of activated sludge biosolids. This is described as bulking and can lead to a release of suspended solids in the treated effluent, a decrease in biosolids concentration in the aeration tank, and poor dewatering-sludge characteristics^1,5^. Bulking is usually caused by excessive proliferation of filamentous bacteria, and their ability to form interfloc bridges, reducing floc settling rates and their compaction, or forming diffuse open floc structures with excess water retention^3^.

Identification of most of the filamentous bacteria responsible for bulking has now been achieved using DNA sequencing advances^4^. For example, the morphotype 1851 recognised by Eikelboom^6^, belongs to the phylum Chloroflexota^7–9^and has been placed conditionally in the genus “*Candidatus* Kouleothrix”^10^. It has been detected in wastewater treatment plant (WWTP) microbialcommunities around the world^11^, but never as a dominant member, with relative abundances of < 1% in every country. However, the authors have reported relative abundances in Japan of > 1%^12,13^ or about 3% on average^14^ for collective members of the genus “*Ca*. Kouleothrix” and with a statistically significant correlation with biomass sludge volume index (SVI).

The full-length database MiDAS 4 with sequences of 16S rRNA gene of activated sludge populations from WWTPs across the world^11^ contains systematic information for members of the Chloroflexota, both cultured and uncultured. In the online MiDAS field guide based on the MiDAS database (https://www.midasfieldguide.org/guide) the *Candidatus* genus *Kouleothrix* is described as embracing 9 species: “*Ca*. K. ribensis” (midas_s_2308)^15^, midas_s_8061, midas_s_25432, midas_s_35412, midas_s_3147, midas_s_3423, midas_s_8041, midas_s_3450, midas_s_1244. In contrast, the Silva database^16^ includes the single isolated and cultured species, *Kouleothrix aurantiaca*^10^. Earlier studies of this filament in WWTP communities have investigated the influence of its seasonal abundance on bulking at the genus level^14,17^. In this study, we describe 16S rRNA targeted fluorescence in situ hybridization (FISH) probes for each of the “*Ca*. Kouleothrix” species identified in a WWTP in Japan^14^ and use these to examine for the first time the environmental, operational, and microbial factors affecting their relative abundances over a 1-year period, and hence their impacts on biomass bulking.

## Materials and methods

### Amplicon sequencing data analysis

In our previous study^14^, 42 activated sludge-mixed liquor samples were collected every 1–2 weeks from an A2O process train, located at a municipal WWTP A in Japan from Sept 2016 to Sept 2017. The settling properties of activated sludge in that train are usually highly seasonally variable and remained so during the study period. The 16S rRNA genes of 12 samples out of the 42 samples were sequenced using their V3–V4 region^14^ and the rawsequence data deposited in the DDBJ Sequence Read Archive (DRA) under the number DRA008490. In this study, the raw sequence data of 11 of the 12 samples were reanalysed using the QIIME2 software package (version 2022.2). The remaining sample was excluded because of the lack of any accompanying SVI data. The raw fastq files were then subjected to a series of filtration and trimming processes referring to the previous study^18^, using Dada2. Generated amplicon sequence variants (ASVs) were assigned against the MiDAS 4 database, Version 4.8.1^11^.

### Phylogenetic analysis and FISH probe design

A maximum-likelihood phylogenetic tree was constructed from full length “*Ca.* Kouleothrix” 16S rRNA gene sequences retrieved from the MiDAS 4 database (version 4.8.1). Oligonucleotide probes targeting “*Ca*. Kouleothrix” species were designed with the probe design/match tools in ARB (ARB-7.0)^19^. Hybridization efficiencies of target and potentially weak non-target matches were assessed in silico with MathFISH web tool^20^ for newly designed probes (KOU83, KOU464, KOU641, and KOU1137; see Table 1). Also, all the four probes designed had ≥ 1.2 mismatch weights (wmis) against the non-target “*Ca*. Kouleothrix” species on the probe design/match tools with the MiDAS 4 database (version 4.8.1). Unlabeled helper probes^21^ were also designed where required. Probes labeled with Cy3 or FAM fluorochromes and unlabeled helper probes were purchased from Eurofins Genomics (Tokyo, Japan) and Fasmac (Atsugi, Japan), respectively.

**Table 1:** Oligonucleotide probes and helper probes used for 16S rRNA targeted FISH probes for “*Ca*. Kouleothrix” species.

Probe name	Target	Morphotype	Sequence (5′−3′)	FA(%)^b^	Reference
KOU83^a^	“*Ca*. Kouleothrix ribensis”	type 1851	CCA CTC AGC ATA GCG AAC	35	This study
KOU464	“*Ca*. Kouleothrix midas_s_1244”	type 1851	CGC GGG GTA CAG TCA CAA	25	This study
KOU641^a^	“*Ca*. Kouleothrix midas_s_3423”	type 1851	CTC TCA AGT CGC GCC GTA	20	This study
KOU1137^a^	“*Ca*. Kouleothrix midas_s_35412”	type 1851	CGT CCG GCC AGA CAG CGT	25	This study
KOU83H1	Helper for KOU83 (Necessary)		TAT GCT GCG TRC GAC TTG CAT GCA		This study
KOU83H2	Helper for KOU83 (Unnecessary)		ACG TGT TCC TCA GCC GTG CG		This study
KOU641H1	Helper for KOU641 (Necessary)		TGC GGT GGC GTC TGG CCG		This study
KOU641H2	Helper for KOU641 (Unnecessary)		CAA TTC CAC GAA CCT CTG		This study
KOU1137H1	Helper for KOU1137 (Unnecessary)		GTA ACT GAC CGT AGG GGT TGC GC		This study
KOU1137H2	Helper for KOU1137 (Necessary)		CCG CCT TCC TCC CRT AAT GGG C		This study

^a^Helper probes are required for the application.

^b^Formamide concentration used.

### FISH analyses

FISH was performed as described previously^22^, essentially following the methods of Daims et al.^23^. To evaluate the formamide (FA) concentrations required for optimum stringency, the newly designed oligonucleotide probes (Table 1) were examined against previously PFA-fixed activated sludge samples^14,22^, changing FA concentration in the hybridization buffer 0–70% in 5% incremental steps. Ten FISH images were taken from each sample at each FA concentration, where fluorescence intensities were measured at five viewpoints with ImageJ software^24^. Mean values and standard deviations were calculated using 50 values of intensity measured for each. Fixed samples were mounted in Vectashield (Vectashield Laboratories, Burlingame, CA) on slide glasses and then observed with an epifluorescence microscope (BX-51, Olympus, Tokyo, Japan) equipped with a CCD camera (DP73, Olympus). The lengths and thicknesses of “*Ca*. Kouleothrix” filaments on FISH images were quantified using a function of the ImageJ software. Mean values, standard deviations, maximum values, and minimum values were calculated based on values of 30 filaments for each sample.

### Statistical analysis

Relationships between the abundance and filament length of “*Ca*. Kouleothrix” species and SVI values were analysed using Pearson correlation analysis for the 11 samples where amplicon sequence raw data (DRA008490) were reanalysed, and for the environmental parameters listed in Supplementary Table S1. Stepwise multiple regression analyses were also performed to confirm how environmental parameters contributed to the abundances and filament lengths of “*Ca*. Kouleothrix” species. All statistical analyses were performed with R v.4.2.1^25^ with statistically significant levels of 5% in the present study.

### Network analysis

Rare ASVs in the data set (those having less than 100 ASVs) were removed. A co-occurrence network was constructed based on the Spearman correlation matrix. Only robust (Spearman’s correlation coefficient |*ρ|* > 0.6) and statistically significant (*p*< 0.05) correlations were considered^26,27^. The resultant correlation matrix was visualised by Cytoscape v.3.10.1.

## Results

### Abundances of “*Ca*. Kouleothrix” species in WWTP A and their correlations with biomass SVI

The genus “*Ca*. Kouleothrix” is considered currently to contain nine distinct species according to the MiDAS 4 taxonomy, six of which (i.e., “*Ca*. K. ribensis”, midas_s_1244, midas_s_3147, midas_s_3423, midas_s_3450, and midas_s_35412) were detected in this study in WWTP A (Supplementary Table S2). Of these midas_s_3423 had the highest relative abundance of 0.879%, followed by midas_s_35412 (0.852%), “*Ca*. K. ribensis” (0.417%), and midas_s_1244 (0.417%). Both midas_s_3147 and midas_s_3450 were not always detected in the samples examined, and thus, their mean abundances were lower than those of the other four species.

**Fig. 1:**
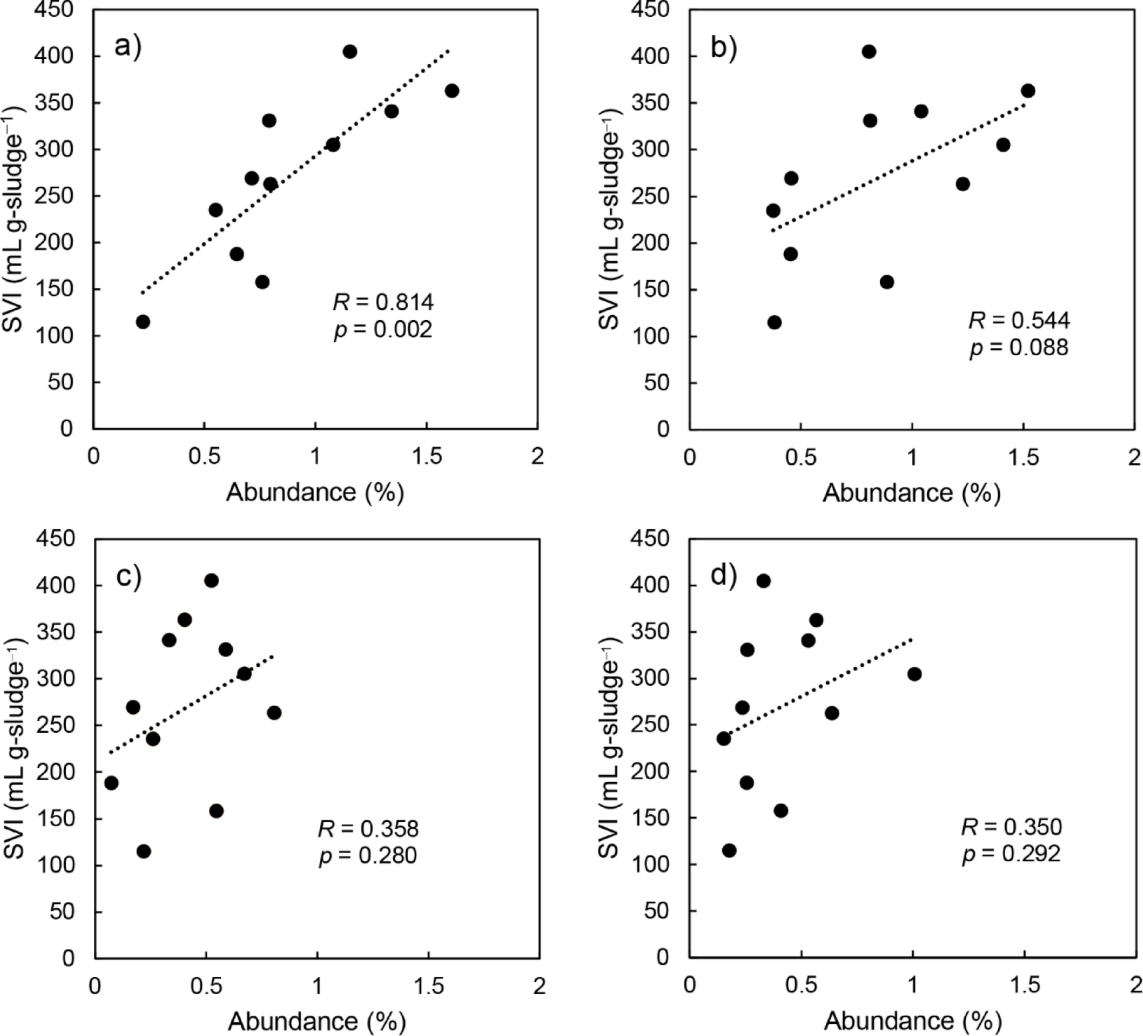
Correlation analyses between relative abundances of “*Ca*. Kouleothrix” species from WWTP A listed in the MiDAS 4 database and SVI (**a**) midas_s_3423, (**b**) midas_s_35412, (**c**) “*Ca*. K. ribensis”, (**d**) midas_s_1244. *R* and *p* show correlation coefficients and *p*-values between relative abundance and SVI, respectively.

We investigated the correlation between biomass SVI and the abundances of each of the “*Ca*. Kouleothrix” species (midas_s_3423, midas_s_35412, “*Ca*. K. ribensis”, and midas_s_1244). Statistical analyses revealed that both midas_s_3423 and midas_s_35412 showed a positive correlation (*R* = 0.814; *p* = 0.002 and *R* = 0.544; *p* = 0.088, respectively) with the corresponding biomass SVI (Fig. 1). Importantly, the correlation of SVI with midas_s_3423 was greater than that with the entire genus (*R* = 0.667; *p* = 0.025, see Supplementary Table S2), indicating its considerable influence on sludge settleability and thus bulking potential.

### Design of FISH based probes targeting each “*Ca*. Kouleothrix” species

**Fig. 2:**
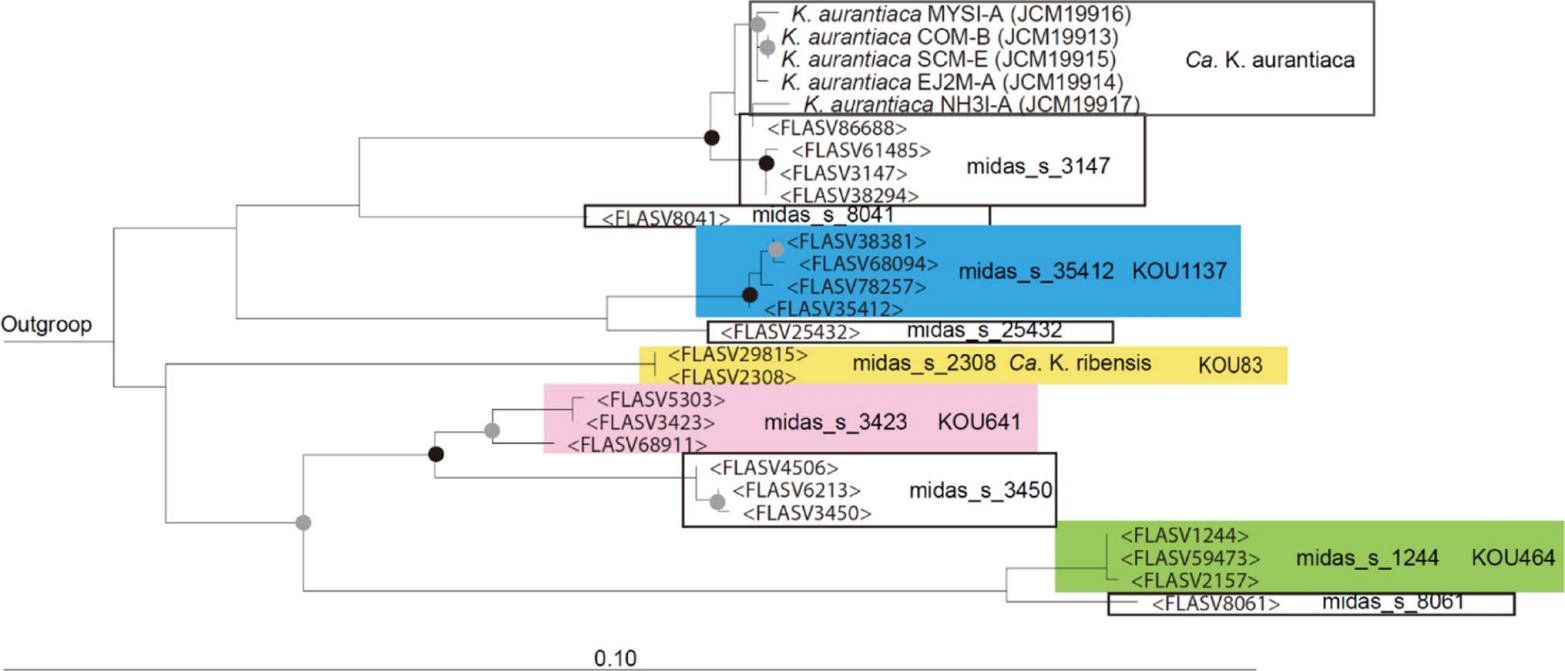
PhyML maximum-likelihood phylogenetic tree was constructed using the full-length of 16S rRNA gene sequences of the cultured and uncultured currently recognised “*Ca*. Kouleothrix”. FISH probe coverages are highlighted by different colors blue (KOU1137), yellow (KOU83), pink (KOU641), and green (KOU464). The branches with over 70% (indicated by gray circles) and 90% (indicated by black circles) support are shown with bootstrap values obtained from 1000 re-samplings. Substitutions per nucleotide base are represented on the scale bar.

We designed four FISH probes KOU83, KOU464, KOU641, and KOU1137 (Table 1), to target “*Ca*. K. ribensis”, midas_s_1244, midas_s_3423, and midas_s_35412 (Fig. 2) populations, respectively. Helper probe sets, KOU83H1 and KOU83H2, KOU641H1 and KOU641H2, and KOU1137H1 and KOU1137H2, were required for the FISH probes KOU83, KOU641, and KOU1137, respectively (Table 1), to increase accessibility to their targets. The fluorescence intensity detected at each FA concentration for probes KOU83, KOU464, KOU641, and KOU1137 was maintained at FA concentrations of 35%, 25%, 20%, and 25%, respectively, but then gradually decreased to 70% (Supplementary Fig. S1). Therefore, these were used in the FISH experiments for the four probes, respectively. Of the six helpers, only KOU83H1, KOU641H1, and KOU1137H2 imparted any enhanced fluorescent signal from their respective targets (Table 1).

### Filament morphology of individual “*Ca*. Kouleothrix” species

**Fig. 3:**
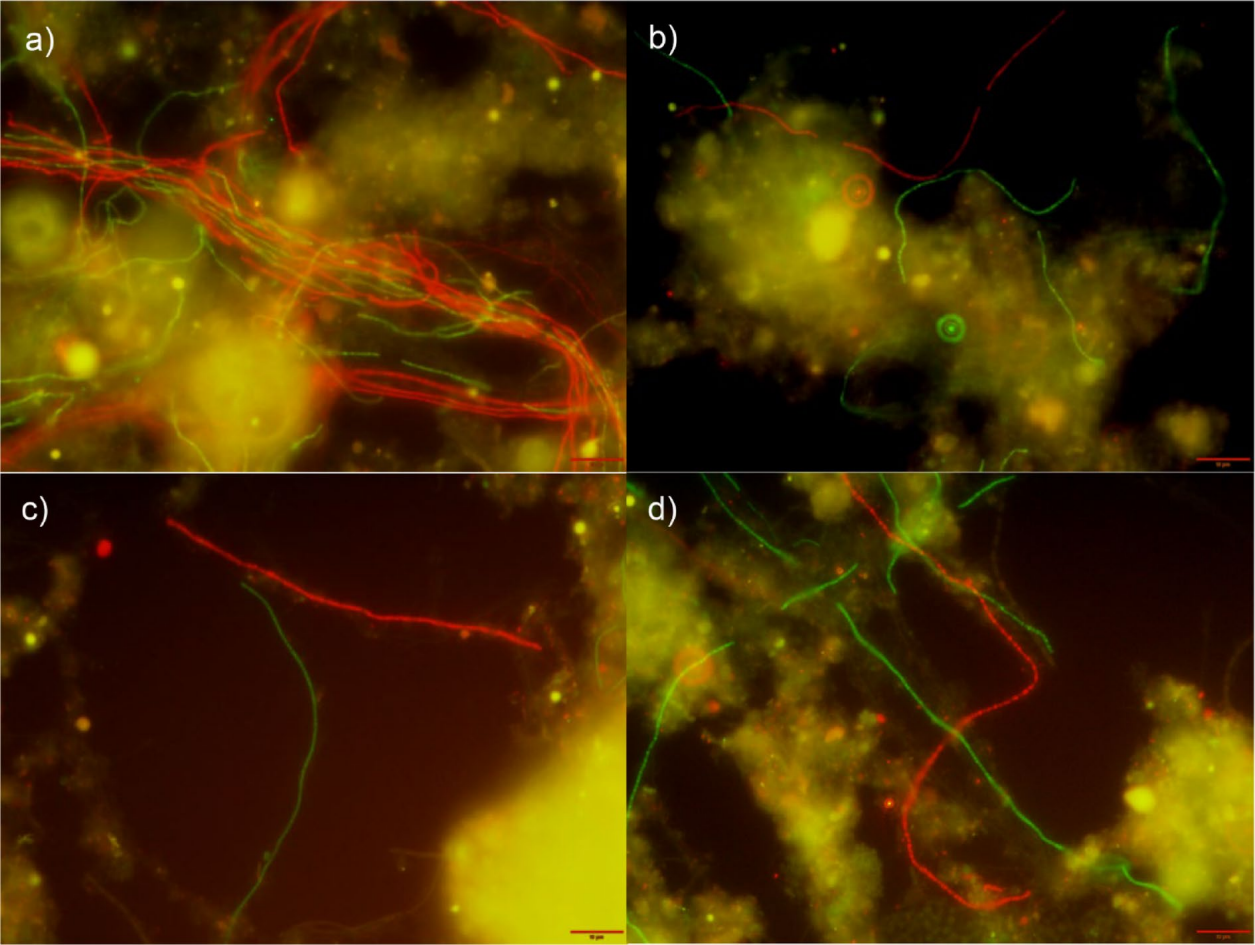
Composite FISH images of “*Ca*. Kouleothrix” species in activated sludge collected from WWTP A. (**a**) midas_s_3423 and midas_s_35412 in bulking sludge (BS: A6 in Table S2) visualised with species-specific probes KOU641 (FAM, green) and KOU1137 (Cy3, red), respectively; (**b**) midas_s_3423 and midas_s_35412 in normal sludge (NS: A9 in Table S2) visualised with KOU641 (FAM, green) and KOU1137 (Cy3, red), respectively; (**c**) “*Ca*. K. ribensis” and midas_s_1244 in BS visualised with species-specific probes KOU83 (FAM, green) and KOU464 (Cy3, red), respectively; (**d**) “*Ca*. K. ribensis” and midas_s_1244 in NS visualised with KOU83 (FAM, green) and KOU464 (Cy3, red), respectively. All scale bars show 10 μm.

**Fig. 4:**
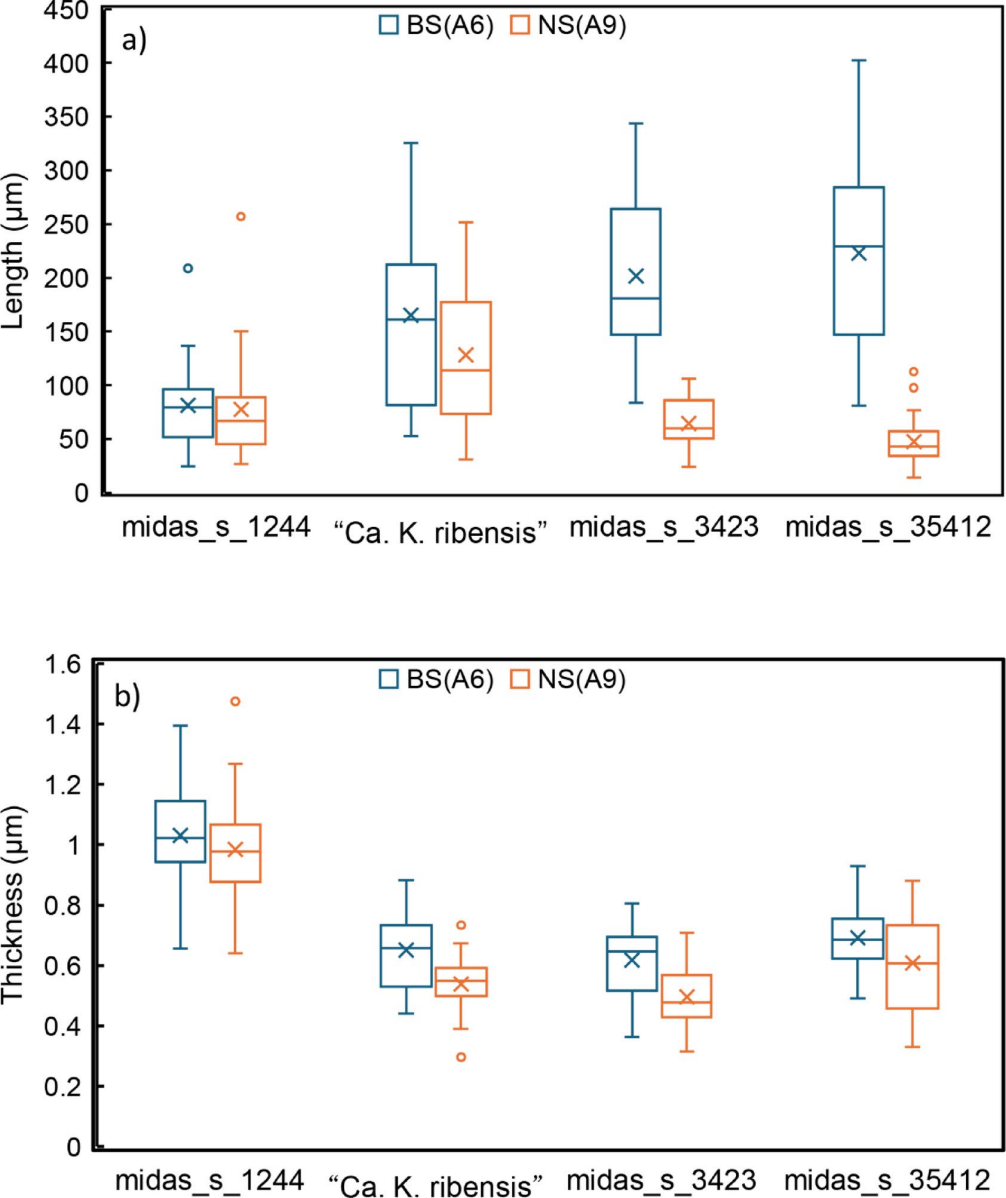
Distribution of filament (**a**) length and (**b**) thickness of each “*Ca*. Kouleothrix” species in both bulking sludge (BS(A6)) and normal sludge (NS(A9)) samples (Table S2). Box plots show 25–75% centiles with min/max whiskers. Crosses in the box plots show mean values.

The morphologies of the four “*Ca*. Kouleothrix” species (“*Ca*. K. ribensis”, midas_s_1244, midas_s_3423, and midas_s_35412) targeted by these new FISH probes (Table 1) were observed in both a bulking sludge (BS: A6 in Supplementary Table S2) and a normal sludge (NS: A9 in Supplementary Table S2). Similar relative abundances of the four were seen, but with large differences in their biomass SVI (Supplementary Table S2). All species shared a curved filiform morphology with no branching (Fig. 3). Interestingly, the two predominant species midas_s_3423 and midas_s_35412 were bundled together in the bulking cables in the bulking sludge BS (Fig. 3a) but separated as short individual filaments in the normal sludge NS (Fig. 3b). There were no obvious changes in filament morphologies of “*Ca*. K. ribensis” and midas_s_1244 in either BS and NS sludges (Fig. 3c, d). Occasionally “*Ca*. K. ribensis” formed cabled bundles, but less commonly than midas_s_3423 and midas_s_35412. Only midas_s_1244 was never seen in this arrangement, possibly because their filaments were much thicker than those of the others. The filament length and thickness of each “*Ca*. Kouleothrix” species in BS and NS are shown in Fig. 4, supporting these descriptions.

### Correlations between filament lengths of “*Ca*. Kouleothrix” and biomass SVI values

**Fig. 5:**
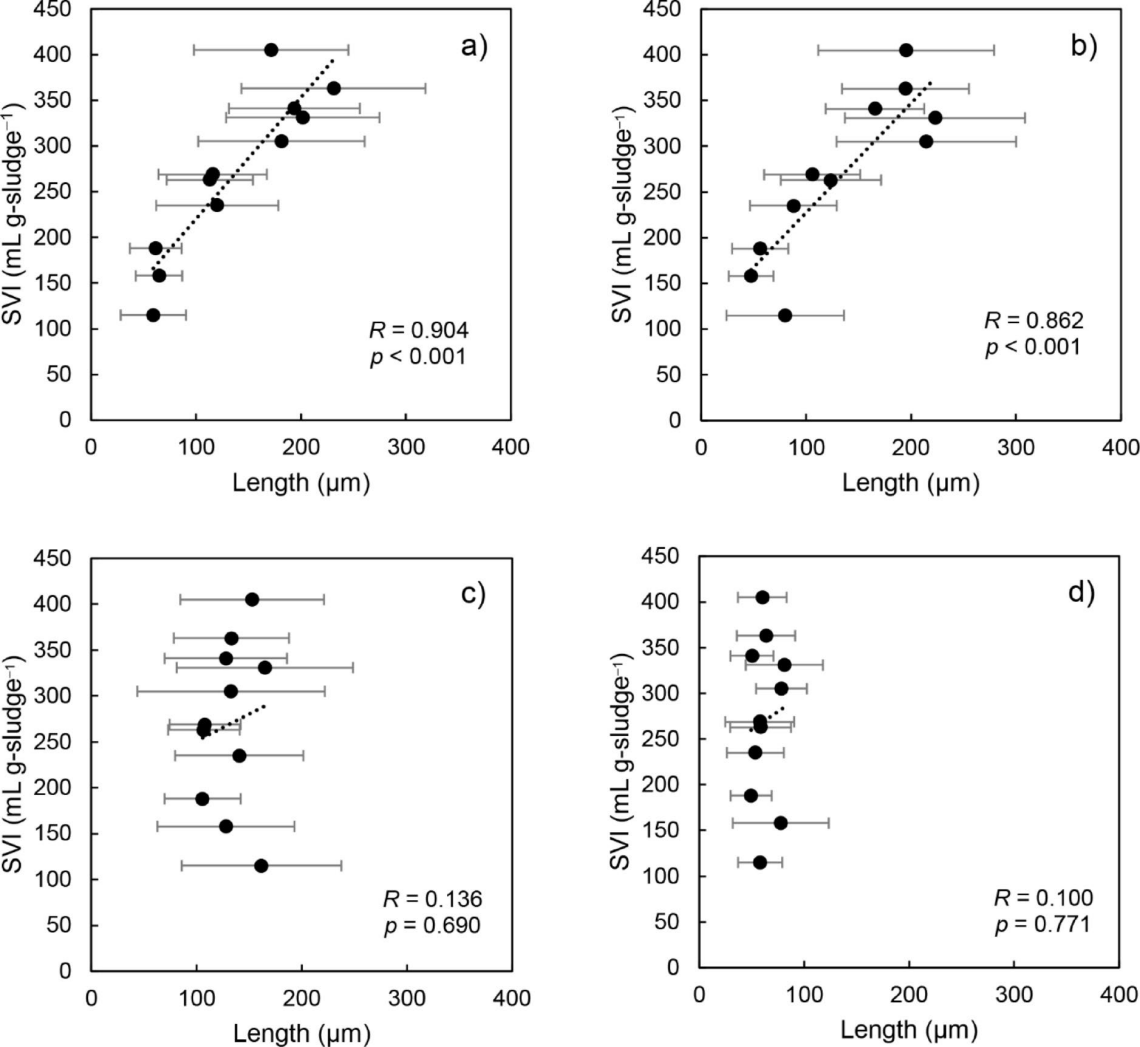
Correlation analysis between filament length of “*Ca*. Kouleothrix” species listed in MiDAS 4 database and SVI (**a**) midas_s_3423, (**b**) midas_s_35412, (**c**) “*Ca*. K. ribensis”, and (**d**) midas_s_1244. *R* and *p* show correlation coefficients and *p*-values, respectively. Error bar indicates the standard deviation of filament length (*n* = 30).

The 11 samples collected from WWTP A (Supplementary Table S3) were examined to see if any correlations between filament lengths of “*Ca*. Kouleothrix” and biomass SVI were present. The result reveals that filament lengths of midas_s_3423 and midas_s_35412 showed statistically significant positive correlations with biomass SVI (Fig. 5a, b, Supplementary Table S3). However, as expected, no similar correlations were seen with “*Ca*. K. ribensis” and midas_s_1244 (Fig. 5c, d, Supplementary Table S3). In fact, filament lengths in both midas_s_3423 and midas_s_35412 correlated more with biomass SVI than with their relative abundances (Fig. 1). Further correlation analyses showed that the abundances of midas_3423 and midas_35412 increased in proportion to their filament lengths (Supplementary Fig. S2a, b), suggesting that both increase their relative abundance values by extending existing filaments, possibly by apical growth, and hence eventually activated sludge bulking occurs. No similar trends were observed in the other two species “*Ca*. K. ribensis” and midas_s_1244 (Supplementary Fig. S2c, d).

### Relationship between “*Ca*. Kouleothrix” and other bacterial populations in WWTP A

To understand how relative abundances of other bacterial populations might relate to the presence of these different “*Ca*. Kouleothrix” species, we conducted Spearman network analyses between them (Supplementary Fig. S3). A total of 37 pairs of statistically significant correlations shown as edges with lines was observed between 22 different bacterial populations including the four “*Ca*. Kouleothrix” spp. (shown as nodes with circles). A total of 34 positive correlations (normal lines) and 3 negative ones (dashed lines) were revealed. “*Ca*. Kouleothrix” midas_s_3423 had the most edges (14 positive and 2 negative edges), followed by “*Ca*. K. ribensis” (10 positive and 1 negative edges), midas_s_35412 (9 positive edges), and midas_s_1244 (5 positive edges). The most and least abundant “*Ca*. Kouleothrix” midas_s_3423 and midas_s_1244 had the widest and narrowest networks respectively. As expected, all four species exhibited high values of local similarity to each other. No correlation was observed between midas_s_3423 and “*Ca*. K. ribensis”. Although midas_s_3423 and “*Ca*. K. ribensis” had 16 and 11 edges, respectively, only five other populations were shown to be commonly correlated with these two, indicating that midas_s_3423 and “*Ca*. K. ribensis” exhibited disparate microbial ecophysiologies. Supplementary Table S4 lists these correlation coefficients and *p*-values for the four “*Ca*. Kouleothrix” species and the other 18 populations.

### Influence of environmental factors on “*Ca*. Kouleothrix”

To explore which operational factors might affect the abundances and filament lengths of midas_s_3423 and midas_s_35412, both showing statistically significant correlation with SVI, multiple regression analysis was conducted. The following parameters suspended solids (SS), biochemical oxygen demand (BOD), total nitrogen (T-N), and total phosphorus (T-P) of effluent from the primary settling tank, mixed liquor temperature, pH, mixed liquor suspended solids (MLSS), dissolved oxygen (DO), hydraulic retention time (HRT), and sludge retention time (SRT) for the aeration tank, and return sludge SS (Supplementary Table S1) were assessed. Pearson correlation analysis was first performed using the abundance/filament lengths of the two species. Then the top five parameters with the highest coefficients listed in Supplementary Table S5 were selected as independent variables for stepwise multiple regression analysis. To account for cases where environmental parameters affected the abundance and filament length of the two species after a certain period, time lagged correlation analyses were also conducted using the operational plant data collected about two weeks before activated sludge samples. The final variable model for the abundances and filament lengths of midas_s_3423 and midas_s_35412 is presented in Eqs. (1–8) below. It was derived using stepwise multiple regression analysis, and both synchronous and lagged correlation. The modified determination coefficient and *p*-value for each equation were as follows: Eq. (1), 0.690, 0.022; Eq. (2), 0.763, 0.023; Eq. (3), 0.740, 0.011; Eq. (4), 0.797, 0.005, Eq. (5), 0.128, 0.237; Eq. (6), 0.854, 0.003; Eq. (7), 0.617, 0.033; Eq. (8), 0.790, 0.002. Supplementary Table S6 also presents both nonstandardised and standardised coefficients, *p*-values, and variance inflation factor (VIF) values.

#### midas_s_3423

##### Synchronous correlations

1 $$\begin{aligned}\:\text{A}\text{b}\text{u}\text{n}\text{d}\text{a}\text{n}\text{c}\text{e}\:\left({\%}\right) & =3.67-1.25 \times\:{10}^{-1}\:\text{Temperature}\:\left(^\circ\text{C}\right)+2.94\times\:{10}^{-1}\:\text{HRT}\:\left(\text{h}\right)\\ & \quad -2.82\times\:{10}^{-1}\:\text{T-N}\:\left(\text{mg}\:{\text{L}}^{-1}\right)\:-4.52\times\:{10}^{-1}\:\text{T-P}\:\left(\text{m}\text{g}\:{\text{L}}^{-1}\right)\end{aligned}$$

2 $$\begin{aligned}\:\text{F}\text{i}\text{l}\text{a}\text{m}\text{e}\text{n}\text{t}\:\text{l}\text{e}\text{n}\text{g}\text{t}\text{h}\:(\upmu\text{m}) &= 6.64 \times 10^{2}- 2.61 \times 10\, \text{Temperature}\, (^\circ \text{C}) + 6.05\,\, \text{SRT (d)} \\ & \quad +3.47 \times 10\, \text{HRT (h)} -3.21 \times 10\, \text{T-N (mg L}^{-1}) \\ & \quad -7.68 \times 10\, \text{T-P (mg L}^{-1}) \end{aligned}$$

##### Lagged correlations

3 $$\begin{aligned}\:\text{A}\text{b}\text{u}\text{n}\text{d}\text{a}\text{n}\text{c}\text{e}\:\left({\%}\right) & =2.68-5.92 \times\:{10}^{-2}\:\text{T}\text{e}\text{m}\text{p}\text{e}\text{r}\text{a}\text{t}\text{u}\text{r}\text{e}\:\left(^\circ\text{C}\right)+1.05\times\:{10}^{-1}\:\text{D}\text{O}\:\left(\text{m}\text{g}\:{\text{L}}^{-1}\right)\\ & \quad -1.24\times\:{10}^{-1}\:\text{T}\text{-}\text{N}\:\left(\text{m}\text{g}\:{\text{L}}^{-1}\right) \end{aligned}$$

4 $$\begin{aligned}\:\text{F}\text{i}\text{l}\text{a}\text{m}\text{e}\text{n}\text{t}\:\text{l}\text{e}\text{n}\text{g}\text{t}\text{h}\:(\upmu\text{m}) & =6.03\times\:{10}^{2}-1.64\times\:10\:\text{T}\text{e}\text{m}\text{p}\text{e}\text{r}\text{a}\text{t}\text{u}\text{r}\text{e}\:\left(^\circ \text{C}\right) -5.18\times\:{10}^{-2}\:\text{M}\text{L}\text{S}\text{S}\:\left(\text{m}\text{g}\:{\text{L}}^{-1}\right) \\ & \quad +1.52\times\:10\:\text{D}\text{O}\:\left(\text{m}\text{g}\:{\text{L}}^{-1}\right) \end{aligned}$$

#### midas_s_35412

##### Synchronous correlations

5 $$\begin{aligned}\text{Abundance}\:\left({\%}\right) &= 1.27-6.48 \times\:{10}^{-2}\:\text{T}\text{e}\text{m}\text{p}\text{e}\text{r}\text{a}\text{t}\text{u}\text{r}\text{e}\:\left(^\circ \text{C}\right)\\ &\quad +\,1.75\times\:{10}^{-4}\,\text{R}\text{e}\text{t}\text{u}\text{r}\text{n}\:\text{s}\text{l}\text{u}\text{d}\text{g}\text{e}\:\text{S}\text{S}\:\left(\text{m}\text{g}\:{\text{L}}^{-1}\right)\end{aligned}$$

6 $$\begin{aligned}\:\text{F}\text{i}\text{l}\text{a}\text{m}\text{e}\text{n}\text{t}\:\text{l}\text{e}\text{n}\text{g}\text{t}\text{h}\:(\upmu\text{m}) & =2.89\times\:{10}^{3}-2.67\times\:10\:\text{T}\text{e}\text{m}\text{p}\text{e}\text{r}\text{a}\text{t}\text{u}\text{r}\text{e}\:\left(^\circ \text{C}\right) \\ & \quad -3.03\times\:{10}^{2}\:\text{p}\text{H}\:\left(-\right)-1.41\times\:{10}^{2}\:\text{T}\text{-}\text{P}\:\left(\text{m}\text{g}\:{\text{L}}^{-1}\right) \\ & \quad -8.60\:\text{S}\text{S}\:\left(\text{m}\text{g}\:{\text{L}}^{-1}\right)\end{aligned}$$

##### Lagged correlations

7 $$\begin{aligned}\:\text{A}\text{b}\text{u}\text{n}\text{d}\text{a}\text{n}\text{c}\text{e}\:\left({\%}\right) & =-7.00\times\:{10}^{-1}+8.30\times\:{10}^{-2}\:\:\text{D}\text{O}\:\left(\text{m}\text{g}\:{\text{L}}^{-1}\right)+4.35\times\:{10}^{-1}\:\text{T}\text{-}\text{P}\:\left(\text{m}\text{g}\:{\text{L}}^{-1}\right) \\ & \quad +1.57\times\:{10}^{-4}\:\text{R}\text{e}\text{t}\text{u}\text{r}\text{n}\:\text{s}\text{l}\text{u}\text{d}\text{g}\text{e}\:\text{S}\text{S}\:\left(\text{m}\text{g}\:{\text{L}}^{-1}\right) \end{aligned}$$

8 $$\:\text{F}\text{i}\text{l}\text{a}\text{m}\text{e}\text{n}\text{t}\:\text{l}\text{e}\text{n}\text{g}\text{t}\text{h}\:(\upmu\text{m})=5.39\times\:{10}^{2}-1.83\times\:10\:\text{T}\text{e}\text{m}\text{p}\text{e}\text{r}\text{a}\text{t}\text{u}\text{r}\text{e}\:\left(^\circ \text{C}\right)+4.26\times\:10\:\text{T}\text{-}\text{P}\:\left(\text{m}\text{g}\:{\text{L}}^{-1}\right)$$

Table 2 presents a summary of the statistically significant factors that influence both the abundances and filament lengths of midas_s_3423 and midas_s_35412, as determined by multiple regression analysis. These data suggest that temperature is the most important negative factor in determining the filament lengths of both midas_s_3423 and midas_s_35412, regardless of whether the correlations are synchronous or lagged. It also appears to be a key factor in the abundance of midas_s_3423 in synchronous correlations. The statistical significance of T-N and DO was observed in both the abundance and filament length of midas_s_3423, in synchronous and lagged correlations, with negative and positive influence respectively. In contrast, pH, T-P, and SS appeared to exert a negative influence onthe filament length of midas_s_35412 in synchronous correlations.

**Table 2:** Statistically significant factors influencing the relative abundances and filament lengths of “*Ca*. Kouleothrix” species; midas_s_3423 and midas_s_35412, as determined by multiple regression analysis in both synchronous and lagged correlations.

MiDAS species	Dependent variable	Synchronous correlations			Lagged correlations		
		Independent variable	Std. coefficient beta	*p*-value	Independent variable	Std. coefficient beta	*p*-value
midas_s_3423	Abundance	Temperature	– 1.00	0.015	DO	0.482	0.046
		T-N	– 0.71	0.016			
	Filament length	Temperature	– 1.00	0.004	Temperature	– 0.815	0.004
		T-N	– 0.51	0.047	DO	0.479	0.025
midas_s_35412	Abundance	N.F.			N.F.		
	Filament length	Temperature	– 1.00	< 0.001	Temperature	– 0.855	< 0.001
		pH	– 0.60	0.007			
		T-P	– 0.97	0.006			
		SS	– 0.42	0.045			

*T-N* Total nitrogen,* T-P* Total phosphorus,* SS*: Suspended solids,* DO* Dissolved oxygen,* N.F.* Not found.

## Discussion

This study has assessed the impact of both biotic and abiotic parameters on the relative abundances of “*Ca*. Kouleothrix”, a filament responsible globally for bulking in activated sludge biomass, and particularly in Japanese WWTP. However, instead of estimating the impact on plant operation of all members within the single genus “*Ca*. Kouleothrix”, this study sought to determine the responses of each of its individual species, as specified in the MiDAS 4 database constructed from global WWTP data^11^ and based on full length 16S rRNA sequences. It required designing species-targeted 16S rRNA-targeted FISH probes for their in situ identification. No prior data were available in MiDAS 4 database on the occurrence of this genus in Japan, since no samples were examined in this global survey from Japanese plants. However, our study showed that six of the nine species listed in MiDAS 4 were present in a single WWTP, with species midas_s_3423 being the most abundant, and midas_s_3147 and midas_s_3450 not always detectable. It also showed a positive correlation between relative abundances and biomass SVI with species midas_s_3423 and midas_s_35412, but not the other species, and with species midas_s_3423 being the strongest, emphasising the importance of population identification to species level in studies of this nature. Furthermore, their relative abundances and responses to a range of plant operational parameters and the presence of other bacterial populations varied considerably, not possible at the genus level.

Results from other similar studies have also been impressive. For example, Nierychlo et al.^18^ showed high abundances globally of a novel previously undescribed species “*Ca*. Microthrix subdominans”, with marked differences in operational importance to the then sole recognised species, “*Ca.* Microthrix parvicella”, known to cause both bulking and foaming. Thus, while “*Ca*. M. parvicella” in WWTPs showed a clear seasonal pattern of abundance, no similar seasonality was detected in “*Ca*. M. subdominans”. Furthermore, “*Ca*. M. parvicella” had a much more substantial impact on the biomass settling properties than did “*C*a. M. subdominans”.

Earlier, Nittami et al.^14^ had reported that members of the genus “*Ca*. Kouleothrix” collectively showed seasonal population dynamics in this same WWTP A, with higher relative abundances occurring in late autumn to early summer, the coldest part of the year. Here seasonal dynamics were examined at the individual species level, and the trends confirm largely their earlier findings. Thus, both midas_s_1244 and “*Ca*. K. ribensis” were more abundant in spring, while midas_s_3423 and midas_s_35412 showed higher abundance over late autumn/winter to spring, and midas_s_3147 abundances were highest in winter. Midas_s_3450 was mainly detectable only in spring (Supplementary Table S2). These data suggest that seasonal dynamics differ between individual species, although confirmation needs this study to be extended to at least 2 years, and with more frequent sampling^28,29^. Nittami et al.^14^ also suggest that “*Ca*. Kouleothrix” were more abundant at cooler temperatures (water temperature range lowest 16.4 and 27.5 °C highest) than in the summer months. This is quite different to the trends seen in most other similar studies, where higher temperatures were considered an important factor in explaining abundance seasonality^29^. When Peces et al.^28^ examined seasonal population dynamics for “*Ca.* Kouleothrix” midas_s_1244 in Danish WWTPs, they identified them as a weakly summer cohort with ‘non-significant’ seasonality, but where mixed liquor temperatures were still low, being between 17 and 19 °C. The equally optimal temperature for the growth of cultured “*Ca*. K. aurantiaca” was determined to be 25–30 °C^10^.

Interestingly filament length in midas_s_3423 and midas_s_35412 followed the same seasonal patterns as relative abundances, with the longest filament lengths seen in winter at low water temperatures (Supplementary Tables S2 and S3). In addition, midas_s_3423 and midas_s_35412 filament lengths both exhibited strong correlations (*R* ≈ 0.9) with SVI values (Fig. 5) close to that of the CMR from a previous study^30^, while “*Ca*. K. ribensis” and midas_s_1244 filament lengths showed no correlation with SVI (*R* ≈ 0.1). Thus, determining lengths of “*Ca*. Kouleothrix” filaments may provide a rapid microscopic indicator of potential activated sludge bulking.

In their survey, Dueholm et al.^11^ reported that the most abundant “*Ca*. Kouleothrix” species globally was midas_s_1244 with mean abundances of 0.002% and 0.078% with V1-V3 and V4 primers, respectively, and while abundances of the individual species varied wildly between plants within and between countries, they were generally the highest (median abundance of 0.002%) in plants designed to remove phosphorus microbiologically. “*Ca. *Kouleothrix” is known to be a facultative anaerobe, with a fermentative metabolism, generating mixed fermentation end products, providing it with a competitive advantage in such treatment plants^9,15^ operating with alternating aerobic: anoxic: anaerobic stages. Their popularity in Japan might also explain why “*Ca. *Kouleothrix” abundances were markedly higher (1.0–4.8%), than in all the other countries surveyed by Dueholm et al^11^.

As already mentioned, no samples were collected in the Dueholm et al.^11^ survey from Japanese plants. Their data again showed large differences within and between the different 30 countries sampled. The highest average “*Ca*. Kouleothrix” relative abundances per plant were observed in Mexico (0.7%), Singapore (0.5%), and Australia (0.4%), while the most abundant species were midas_s_3423 (in the EBPR process in Mexico and the carbon removal process in Australia), midas_s_1244 (in the EBPR process in Australia), and midas_s_3147 (in the denitrification process in Australia), while midas_s_1244 was the prevalent species in long-term surveys of over 20 Danish full-scale BNR plants, with a median abundance of 0.013%, followed by midas_s_3423 (median abundance of 0.001%).

However, it is not known which operating conditions and environmental parameters might determine the relative abundances of each individual species in each plant^11^. Nor is the impact of filament length or organization (individual or cable) on bulking, well understood, although this study shows, like Liao et al.^30^, that “*Ca*. Kouleothrix” species, some more than others, can extend filaments as they proliferate, thereby increasing and strengthening interfloc bridging, especially if in cabled form. Furthermore, according to the genome data deposited in NCBI, the copy number of the 16S rRNA gene of “*Ca*. Kouleothrix” is between 1 and 3 (Acc. JADKGW01, JAXLWP01, JAXLWQ01, and LJCR01), which is lower than that of the bacterial average of 5.3 ± 2.8^31^. If this is the case in all the species in “*Ca*. Kouleothrix”, the actual relative abundance of this group should be higher than that estimated with amplicon sequencing. Although we have no information about their cell size except for the diameter (between 0.6 and 0.8 μm)^32^, if the cells of this group are larger than others, even a relatively small abundance could correspond to a larger biomass responsible for bulking.

Finally, what this study does show is that in any attempt to control “*Ca. *Kouleothrix” bulking should be targeted and based on identifying which of the MiDAS 4 species are responsible. Based on the extent of the Dueholm et al.^11^ global survey, it seems unlikely although not impossible that other additional “*Ca.* Kouleothrix” species might exist in other habitats and knowing how they differ from each other ecologically might provide clues for a selective approach to bulking control.

## Conclusions

This study focused on the responsibility of “*Ca*. Kouleothrix” at the species level in the phenomenon of activated sludge bulking. The use of newly developed species-specific FISH probes revealed that filament length was dependent on species and abundance, with statistically significant correlations observed between this variation and SVI. Correlation analyses using amplicon sequencing data also indicated the relative abundance of each species in the presence of biotic and abiotic factors. These approaches enabled the classification of species responsible and unresponsible for bulking events and provided insights into potential bulking control strategies. While further studies with longer time series at multiple WWTPs are necessary to gain a deeper understanding for “*Ca*. Kouleothrix”, this research highlights the value of species-level analysis in this field.

## Electronic supplementary material

Below is the link to the electronic supplementary material.

Supplementary Material 1.

## Data Availability

The datasets generated during and/or analysed during the current study are available from the corresponding author on reasonable request. The raw sequence data deposited in the DDBJ Sequence Read Archive (DRA) under the number DRA008490 (https://ddbj.nig.ac.jp/search/entry/sra-submission/DRA008490).
