# Supplementary material for: The impact of biotic and abiotic interactions on Candidatus Kouleothrix bulking in a full-scale activated sludge anaerobic-anoxic-oxic plant in Japan
Source: Sci Rep. 2025 May 6;15:15842. doi: 10.1038/s41598-025-98211-9 (PMC12056232; doi:10.1038/s41598-025-98211-9)
Supplement: Supplementary file 1 — Supplementary Material 1. [file 41598_2025_98211_MOESM1_ESM.pdf]

The impact of biotic and abiotic interactions on *Candidatus* Kouleothrix bulking in a full-scale activated sludge anaerobic-anoxic-oxic plant in Japan

Tadashi Nittami [a,*], Nagi Ishizuka [b], Yoshiki Sakurai [c], Robert J Seviour [d]

[a] Division of Materials Science and Chemical Engineering, Faculty of Engineering, Yokohama National University, 79-5 Tokiwadai, Hodogaya-ku, Yokohama 240-8501, Japan

[b] Department of Chemistry and Life Science, Graduate School of Engineering Science, Yokohama National University, 79-5 Tokiwadai, Hodogaya-ku, Yokohama 240-8501, Japan

[c] Division of Artificial Environment and Information, Faculty of Environment and Information Sciences, Yokohama National University, 79-7 Tokiwadai, Hodogaya-ku, Yokohama 240-8501, Japan

[d] Department of Physiology, Anatomy, and Microbiology, La Trobe University, Bundoora, VIC3086, Australia

[*] Corresponding author at: Division of Materials Science and Chemical Engineering, Faculty of Engineering, Yokohama National University, 79-5 Tokiwadai, Hodogaya-ku, Yokohama 240-8501, Japan. Tel.: +81 45 339 4006

E-mail address: nittami@ynu.ac.jp (T. Nittami).

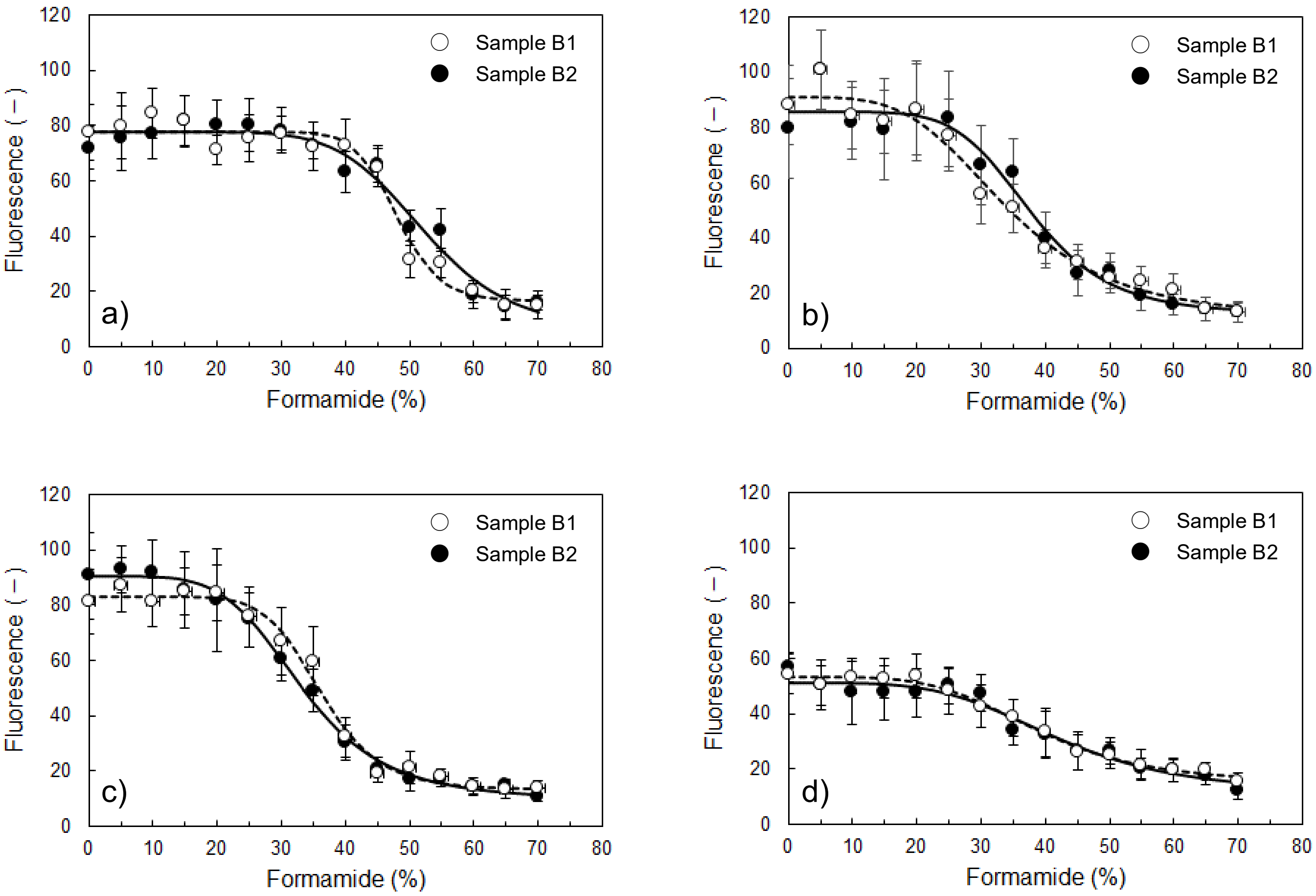

Fig. S1 Dissociation curves determined for four oligonucleotide probes targeting "*Ca*. Kouleothrix" MiDAS 4 species, showing how formamide concentrations affect fluorescence emission values of each FISH probe a) KOU83, b) KOU464, c) KOU641, and d) KOU1137 against two PFA-fixed activated sludge samples collected from different trains in WWTP B. Mean values (symbol) and standard deviations (error bar) were calculated using 50 values (n = 50) of intensity measured for each.

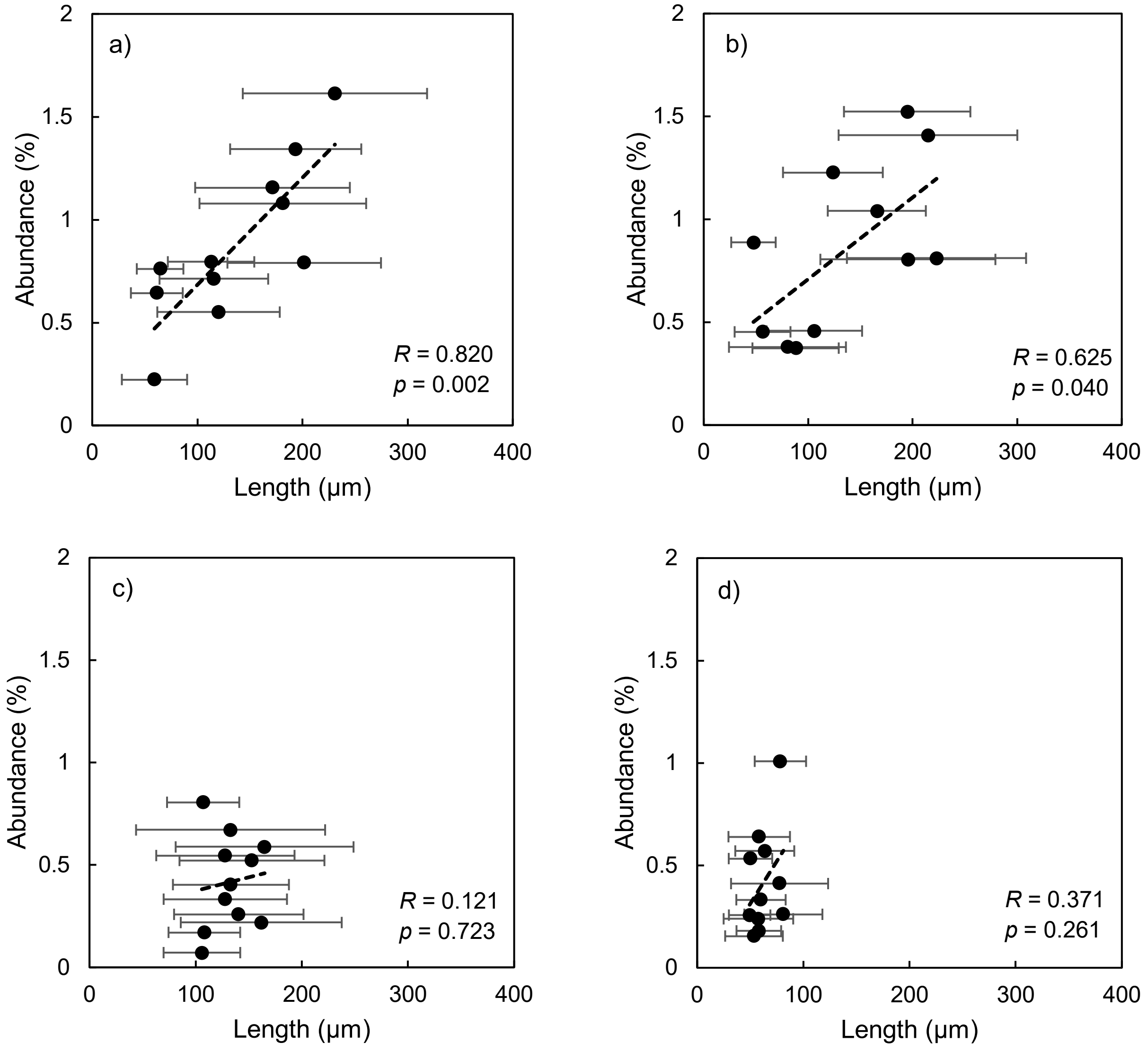

Fig. S2 Correlation analysis between relative abundance and filament length of "*Ca*. Kouleothrix" MiDAS 4 species. a) midas_s_3423, b) midas_s_35412, c) "*Ca*. K. ribensis", and d) midas_s_1244. *R* and *p* show correlation coefficients and *p*-values, respectively. Error bar indicates the standard deviation of filament length ($n = 30$).

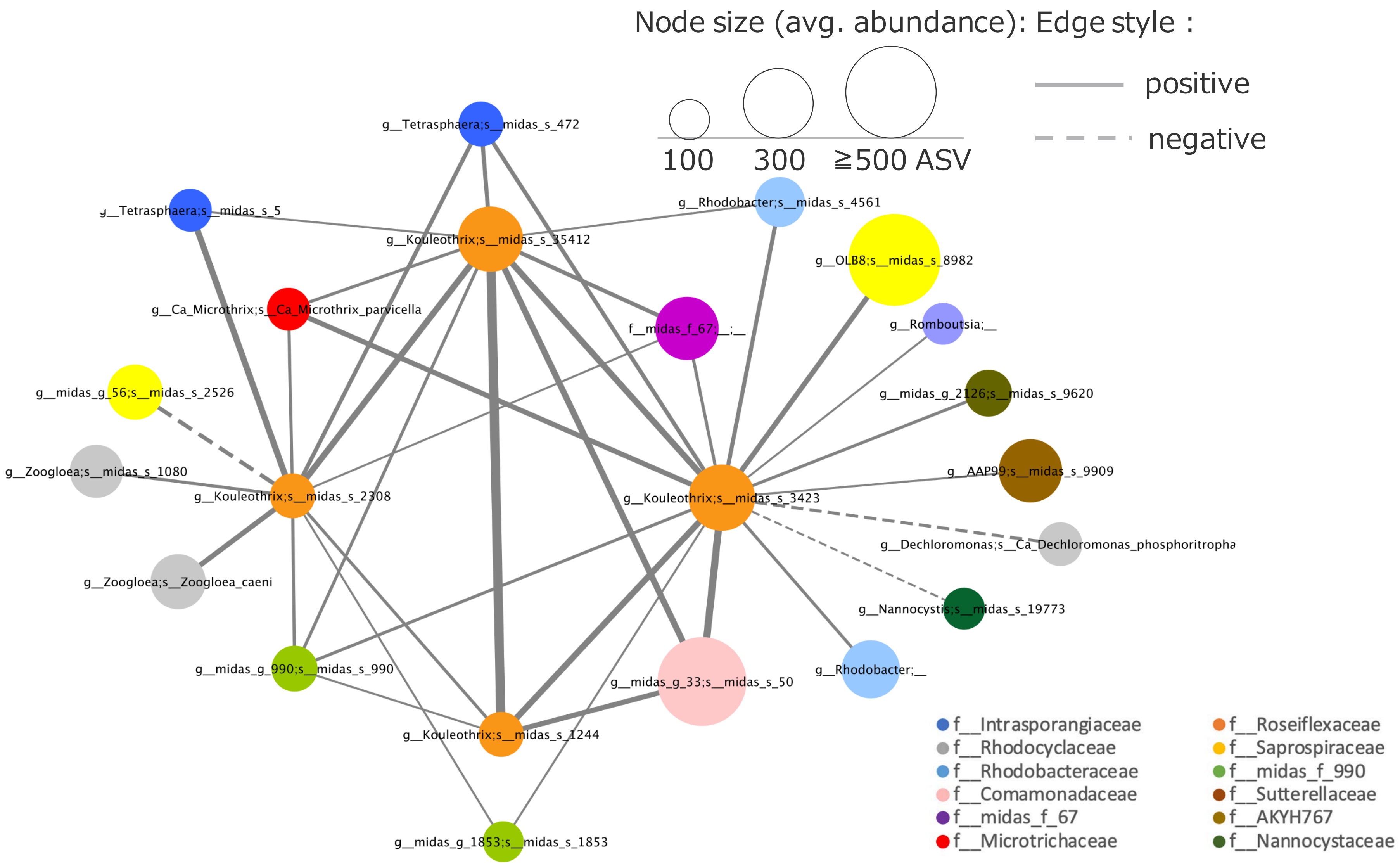

Fig. S3 Spearman network analyses showing relationship between "*Ca.* Kouleothrix" spp. and other populations in WWTP A. The node represents an amplicon sequence variant (ASV), with color and size indicating family and average number, respectively. Edge thickness and style indicate proportionality to the absolute value of local similarity and positive/negative correlation, respectively.

Table S1 Environmental parameter data for the A2O process train from 11 sludge samples used for statistical analyses.

	**Temp [a] [°C]**	**pH [a] [−]**	**MLSS [a] [mg L^{-1}]**	**DO [a] [mg L^{-1}]**	**SRT [a] [d]**	**HRT [a] [d]**	**SS [b] [mg L^{-1}]**	**BOD [b] [mg L^{-1}]**	**T-N [b] [mg L^{-1}]**	**T-P [b] [mg L^{-1}]**	**SS [c] in return sludge [mg L^{-1}]**
**Range**	16.4–27.5	6.5–6.9	1600–2640	1.7–6.4	5.9–19.5	6.5–8.2	19–35	34.5–95.6	15.9–28.4	1.38–4.17	3900–7730
**Average**	23.3	6.6	2292	2.5	12.4	7.34	28	69.5	23.3	2.43	6247
**S.D.**	3.49	1.00×10^{-1}	293.2	1.3	4.05	5.47×10^{-1}	4.4	15.1	3.65	7.39×10^{-1}	1207

[a] Parameters assessed in aeration tank samples. [b] Parameters assessed in effluent water samples from the primary settlement tank (i.e., influent water into anaerobic tank). [c] Parameters assessed in circulated return sludge samples from the secondary settlement tank to anaerobic tank. Parameters measured BOD: Biochemical oxygen demand; T-N: Total nitrogen; T-P: Total phosphorus; SS: Suspended solids; SVI: Sludge volume index; DO: Dissolved oxygen; MLSS: Mixed liquor suspended solids; SRT: Sludge retention time; HRT: Hydraulic retention time.

Table S2 Relative abundances of 16S rRNA gene of "*Ca*. Kouleothrix" spp. in activated sludge samples from 11 days and its correlation with biomass SVI.

**Sample (date)**	**A1 (26/9)**	**A2 (24/10)**	**A3 (21/11)**	**A4 (5/12)**	**A5 (10/1)**	**A6 (20/2)**	**A7 (1/6)**	**A8 (29/6)**	**A9 (13/7)**	**A10 (27/7)**	**A11 (8/9)**	**Ave. (A:A1-A3)** [b]	**Ave. (W:A4 -A6)** [b]	**Ave. (Sp:A7 -A8)** [b]	**Ave. (Su:A9 -A11)** [b]	**Ave. (Y:A1-A11)** [b]	***R*** [c]	***p*** [c]
**midas_s_1244**	0.257	0.239	0.533	0.57	0.332	0.261	1.008	0.64	0.411	0.18	0.155	0.343 ± 0.165	0.388 ± 0.162	0.824 ± 0.260	0.249 ± 0.141	0.417 ± 0.255	0.350	0.292
**"*Ca*. K. ribensis"**	0.072	0.171	0.333	0.404	0.522	0.588	0.671	0.806	0.546	0.219	0.259	0.192 ± 0.132	0.505 ± 0.093	0.739 ± 0.095	0.341 ± 0.178	0.417 ± 0.229	0.358	0.28
**midas_s_3147**	0	0.037	0	0.628	0.365	0.261	0.253	0.159	0	0	0.025	0.012 ± 0.021	0.418 ± 0.189	0.206 ± 0.066	0.008 ± 0.014	0.157 ± 0.204	N.D.	N.D.
**midas_s_3423**	0.644	0.712	1.343	1.613	1.156	0.79	1.079	0.795	0.761	0.223	0.551	0.900 ± 0.385	1.186 ± 0.412	0.937 ± 0.201	0.512 ± 0.271	0.879 ± 0.390	0.814	0.002
**midas_s_3450**	0	0	0	0.019	0	0	0.135	0.069	0.065	0	0	0 ± 0	0.006 ± 0.011	0.102 ± 0.047	0.022 ± 0.038	0.026 ± 0.045	N.D.	N.D.
**midas_s_35412**	0.453	0.457	1.041	1.523	0.805	0.811	1.409	1.228	0.888	0.379	0.374	0.650 ± 0.338	1.046 ± 0.413	1.319 ± 0.128	0.547 ± 0.295	0.852 ± 0.414	0.544	0.088
**"*Ca*. Kouleothrix"** [a]	1.426	1.617	3.25	4.756	3.211	2.711	4.554	3.697	2.671	1.001	1.364	2.098 ± 1.003	3.559 ± 1.066	4.126 ± 0.606	1.679 ± 0.878	2.751 ± 1.291	0.667	0.025
**SVI (mL g^{-1})**	188	269	341	363	405	331	305	263	158	115	235	266 ± 77	366 ± 37	284 ± 30	169 ± 61	270 ± 90		

[a] "*Ca*. Kouleothrix" species in this genus, from which the data for genus-level analysis are given. [b] Average (Ave.) shows the mean values of the biomass abundances and SVIs with standard deviation from 3 samples (A1-A3) in autumn (A), 3 samples (A4-A6) in winter (W), 2 samples (A7-A8) in spring (Sp), 3 samples (A9-A11) in summer (Su), and 11 samples (A1-A11) in a year (Y). [c] *R* and *p* show correlation coefficients and *p*-values between abundance and biomass SVI, respectively. N.D. means not determined.

Table S3 Filament lengths (µm) of "*Ca*. Kouleothrix" spp. in activated sludge samples from 11 days and its correlation with biomass SVI.

**Sample (date)**	**A1 (26/9)**	**A2 (24/10)**	**A3 (21/11)**	**A4 (5/12)**	**A5 (10/1)**	**A6 (20/2)**	**A7 (1/6)**	**A8 (29/6)**	**A9 (13/7)**	**A10 (27/7)**	**A11 (8/9)**	**Ave. (A:A1-A3)**[a]	**Ave. (W:A4 -A6)**[a]	**Ave. (Sp:A7 -A8)**[a]	**Ave. (Su:A9 -A11)**[a]	**Ave. (Y:A1-A11)**[a]	***R*** [b]	***p*** [b]
**midas_s_1244**	49.41	57.67	50.18	63.65	60.09	81.17	78.36	58.42	77.59	58.05	53.50	52.42 ± 4.56	68.30 ±11.29	68.39 ±14.11	63.05 ±12.80	62.55 ±11.40	0.100	0.771
**"*Ca*. K. ribensis"**	105.89	108.20	127.93	133.21	153.04	164.94	132.91	107.07	128.01	161.77	140.64	114.01 ±12.11	150.40 ±16.03	119.99 ±18.27	143.47 ±17.06	133.05 ±20.93	0.136	0.690
**midas_s_3423**	61.43	115.68	193.52	230.83	171.46	201.62	181.27	113.01	64.54	59.21	120.12	123.54 ±66.40	201.30 ±29.68	147.14 ±48.27	81.29± 33.73	137.52 ±61.36	0.904	<0.001
**midas_s_35412**	56.28	105.79	165.60	194.66	195.35	222.72	214.56	123.59	47.58	80.11	87.90	109.22 ±54.74	204.24 ±16.01	169.07 ±64.32	71.86± 21.39	135.83 ±64.97	0.862	<0.001
**SVI (mL g^{-1})**	188	269	341	363	405	331	305	263	158	115	235	266 ±77	366 ±37	284 ±30	169 ±61	270 ±90		

[a] Average (Ave.) shows the mean values of the filament length and SVIs with standard deviation from 3 samples (A1-A3) in autumn (A), 3 samples (A4-A6) in winter (W), 2 samples (A7-A8) in spring (Sp), 3 samples (A9-A11) in summer (Su), and 11 samples (A1-A11) in a year (Y). [b] *R* and *p* show correlation coefficients and *p*-values between filament length and biomass SVI, respectively.

Table S4 Population showing statistically significant correlation with more than one of the four "*Ca*. Kouleothrix" species in Spearman network analysis (top row: correlation coefficient; bottom row in parenthesis: *p*-value). Grey background indicates statistically insignificant correlations ($p > 0.05$), which are not shown in **Fig. S3** as edges. The list is ordered in decreasing ASV value.

Species	ASV	midas_s_3423	midas_s_35412	"*Ca.* K. ribensis"	midas_s_1244
**f__Saprospiraceae;g__OLB8;s__midas_s_8982**	502	0.765<br>(0.006)	0.373<br>(0.259)	0.050<br>(0.884)	0.409<br>(0.212)
**f__Comamonadaceae;g__midas_g_33;s__midas_s_50**	470	0.888<br>(<0.001)	0.800<br>(0.003)	0.579<br>(0.062)	0.782<br>(0.004)
**f__Sutterellaceae;g__AAP99;s__midas_s_9909**	240	0.633<br>(0.036)	0.300<br>(0.370)	0.005<br>(0.989)	0.236<br>(0.484)
**f__midas_f_67;__;__**	238	0.697<br>(0.017)	0.700<br>(0.016)	0.606<br>(0.048)	0.573<br>(0.066)
**f__Rhodobacteraceae;g__Rhodobacter;__**	202	0.661<br>(0.027)	0.364<br>(0.272)	−0.050<br>(0.884)	0.291<br>(0.386)
**f__Rhodocyclaceae;g__Zoogloea;s__Zoogloea_caeni**	182	0.247<br>(0.465)	0.410<br>(0.210)	0.795<br>(0.003)	0.278<br>(0.408)
**f__Saprospiraceae;g__midas_g_56;s__midas_s_2526**	182	−0.041<br>(0.905)	−0.373<br>(0.259)	−0.724<br>(0.012)	−0.309<br>(0.355)
**f__Rhodocyclaceae;g__Zoogloea;s__midas_s_1080**	171	−0.032<br>(0.926)	0.218<br>(0.519)	0.679<br>(0.022)	0.100<br>(0.770)
**f__Rhodobacteraceae;g__Rhodobacter;s__midas_s_4561**	148	0.747<br>(0.008)	0.647<br>(0.031)	0.461<br>(0.153)	0.501<br>(0.116)
**f__AKYH767;g__midas_g_2126;s__midas_s_9620**	133	0.683<br>(0.020)	0.564<br>(0.071)	0.415<br>(0.205)	0.409<br>(0.212)
**f__midas_f_990;g__midas_g_990;s__midas_s_990**	124	0.693<br>(0.018)	0.680<br>(0.021)	0.654<br>(0.029)	0.626<br>(0.040)
**f__Intrasporangiaceae;g__Tetrasphaera;s__midas_s_472**	120	0.705<br>(0.015)	0.747<br>(0.008)	0.744<br>(0.009)	0.560<br>(0.073)
**f__Rhodocyclaceae;g__Dechloromonas;s__Ca_Dechloromonas_phosphoritropha**	115	−0.661<br>(0.027)	−0.327<br>(0.326)	0.046<br>(0.894)	−0.245<br>(0.467)
**f__Intrasporangiaceae;g__Tetrasphaera;s__midas_s_5**	111	0.392<br>(0.233)	0.645<br>(0.032)	0.806<br>(0.003)	0.482<br>(0.133)
**f__Microtrichaceae;g__Ca_Microthrix;s__Ca_Microthrix_parvicella**	109	0.765<br>(0.006)	0.658<br>(0.028)	0.664<br>(0.026)	0.505<br>(0.113)
**f__Nannocystaceae;g__Nannocystis;s__midas_s_19773**	105	−0.624<br>(0.040)	−0.491<br>(0.125)	−0.351<br>(0.290)	−0.364<br>(0.272)
**f__Peptostreptococcaceae;g__Romboutsia;__**	104	0.650<br>(0.030)	0.452<br>(0.163)	0.119<br>(0.728)	0.457<br>(0.158)
**f__midas_f_990;g__midas_g_1853;s__midas_s_1853**	101	0.606<br>(0.048)	0.509<br>(0.110)	0.606<br>(0.048)	0.427<br>(0.190)

Table S5 Correlation coefficients both in synchronous and lagged correlation between environmental factors and the abundance and filament lengths of midas_s_3423 and midas_s_35412.

	**midas_s_3423**				**midas_s_35412**			
	**Synchronous correlation**		**Lagged correlation**		**Synchronous correlation**		**Lagged correlation**	
	**Abundance**	**Filament length**	**Abundance**	**Filament length**	**Abundance**	**Filament length**	**Abundance**	**Filament length**
**Temperature**	–0.659	–0.749	–0.621	–0.811	–0.299	–0.751	–0.438	–0.868
**pH**	0.101	–0.271	0.343	0.164	–0.099	–0.463	0.178	–0.189
**MLSS**	0.193	0.234	0.431	0.361	0.332	0.171	0.499	0.454
**DO**	0.161	0.080	0.739	0.614	–0.120	0.212	0.571	0.349
**SRT**	–0.499	–0.307	–0.617	–0.610	–0.053	–0.196	–0.122	–0.495
**HRT**	–0.455	–0.426	–0.481	–0.484	0.034	–0.285	–0.042	–0.195
**BOD**	–0.311	–0.064	–0.510	–0.013	–0.326	0.011	–0.582	0.144
**T-N**	–0.616	–0.352	–0.611	–0.225	–0.362	–0.108	–0.545	–0.101
**T-P**	0.383	0.355	0.241	0.249	–0.004	0.373	0.608	0.326
**SS**	–0.188	0.167	–0.582	–0.190	–0.088	0.326	–0.493	–0.192
**Return sludge SS**	–0.108	–0.054	0.346	–0.087	0.243	–0.037	0.649	–0.138

BOD: Biochemical oxygen demand; T-N: Total nitrogen; T-P: Total phosphorus; SS: Suspended solids; SVI: Sludge volume index; DO: Dissolved oxygen; MLSS: Mixed liquor suspended solids; SRT: Sludge retention time; HRT: Hydraulic retention time.

Table S6 List of coefficients, *p*-values, and VIF values pertaining to the final multiple regression model between the relative abundances and filament lengths of "*Ca.* Kouleothrix" species midas_s_3423 and midas_s_35412 and environmental parameters, both after synchronous and lagged correlation analyses.

MiDAS species	Correlation	Dependent variable	Independent variable	Unstd. Coefficient		Std. coefficient beta	*p*-value	VIF
				B	Std. error			
midas_s_3423	Synchronous	Abundance	Constant	3.67	1.03			
			Temperature	-1.25×10^{-1}	3.69×10^{-2}	–1.00	0.015	3.969
			HRT	2.94×10^{-1}	1.82×10^{-1}	0.43	0.157	2.459
			T-N	-2.82×10^{-1}	8.52×10^{-2}	–0.71	0.016	1.599
			T-P	-4.52×10^{-1}	2.54×10^{-1}	–0.52	0.126	3.170
		Filament length	Constant	6.64×10^{2}	1.46×10^{2}			
			Temperature	-2.61×10	5.14	–1.00	0.004	3.969
			SRT	6.05	3.68	0.42	0.161	2.725
			HRT	3.47×10	2.59×10	0.32	0.237	2.459
			T-N	-3.21×10	1.22×10	–0.51	0.047	1.599
			T-P	-7.68×10	3.75×10	–0.56	0.096	3.170
	Lagged	Abundance	Constant	2.68	0.74			
			Temperature	-5.92×10^{-2}	2.44×10^{-2}	–0.430	0.052	2.504
			DO	1.05×10^{-1}	4.17×10^{-2}	0.482	0.046	1.317
			T-N	-1.24×10^{-1}	7.02×10^{-2}	–0.333	0.127	2.368
		Filament length	Constant	6.03×10^{2}	1.51×10^{2}			
			Temperature	-1.64×10	3.62	–0.815	0.004	3.181
			MLSS	-5.18×10^{-2}	3.83×10^{-2}	–0.250	0.225	1.919
			DO	1.52×10	5.14	0.479	0.025	1.628
midas_s_35412	Synchronous	Abundance	Constant	1.27	7.54×10^{-1}			
			Temperature	-6.48×10^{-2}	1.21×10^{-1}	–0.57	0.134	1.926
			Return sludge SS	1.75×10^{-4}	1.12×10^{-4}	0.54	0.157	7.992
		Filament length	Constant	2.89×10^{3}	5.57×10^{3}			
			Temperature	-2.67×10	4.14	–1.00	<0.001	6.298
			pH	-3.03×10^{2}	7.51×10	–0.60	0.007	4.174
			T-P	-1.41×10^{2}	3.37×10	–0.97	0.006	3.700
			SS	–8.60	3.41	–0.42	0.045	1.907
	Lagged	Abundance	Constant	-7.00×10^{-1}	5.39×10^{-1}			
			DO	8.30×10^{-2}	4.88×10^{-2}	0.373	0.140	1.607
			T-P	4.35×10^{-1}	2.10×10^{-1}	0.445	0.083	1.467
			Return sludge SS	1.57×10^{-4}	8.76×10^{-5}	0.403	0.124	6.140
		Filament length	Constant	5.39×10^{2}	7.88×10			
			Temperature	-1.83×10	3.28	–0.855	<0.001	4.987
			T-P	4.26×10	2.27×10	0.288	0.102	2.133

T-N: Total nitrogen; T-P: Total phosphorus; SS: Suspended solids; DO: Dissolved oxygen; MLSS: Mixed liquor suspended solids; SRT: Sludge retention time; HRT: Hydraulic retention time.
